# Co-delivery of micronized urinary bladder matrix damps regenerative capacity of minced muscle grafts in the treatment of volumetric muscle loss injuries

**DOI:** 10.1371/journal.pone.0186593

**Published:** 2017-10-17

**Authors:** Stephen M. Goldman, Benjamin T. Corona

**Affiliations:** United States Army Institute of Surgical Research, Fort Sam Houston, TX, United States of America; University of Minnesota Medical Center, UNITED STATES

## Abstract

Minced muscle grafts (MG) promote de novo muscle fiber regeneration and neuromuscular strength recovery in small and large animal models of volumetric muscle loss. The most noteworthy limitation of this approach is its reliance on a finite supply of donor tissue. To address this shortcoming, this study sought to evaluate micronized acellular urinary bladder matrix (UBM) as a scaffolding to promote *in vivo* expansion of this MG therapy in a rat model. Rats received volumetric muscle loss injuries to the tibialis anterior muscle of their left hind limb which were either left untreated or repaired with minced muscle graft at dosages of 50% and 100% of the defect mass, urinary bladder matrix in isolation, or a with an expansion product consisting of a combination of the two putative therapies in which the minced graft is delivered at a dosage of 50% of the defect mass. Rats survived to 2 and 8 weeks post injury before functional (in vivo neuromuscular strength), histological, morphological, and biochemical analyses were performed. Rats treated with the expansion product exhibited improved neuromuscular function relative to untreated VML after an 8 week time period following injury. This improvement in functional capacity, however, was accompanied with a concomitant reduction in graft mediated regeneration, as evidenced cell lineage tracing enable by a transgenic GFP expressing donor, and a mixed histological outcome indicating coincident fibrous matrix deposition with interspersed islands of nascent muscle fibers. Furthermore, quantitative immunofluorescence and transcriptional analysis following the 2 week time point suggests an exacerbated immune response to the UBM as a possible nidus for the observed suboptimal regenerative outcome. Moving forward, efforts related to the development of a MG expansion product should carefully consider the effects of the host immune response to candidate biomaterials in order to avoid undesirable dysregulation of pro-regenerative cross talk between the immune system and myogenic processes.

## Background

Battlefield extremity injuries often give rise to large skeletal muscle defects either as the direct result of trauma or surgical procedures, such as fasciotomy and debridement, throughout the continuum of care [1]. Because there is no standard of care, volumetric muscle loss (VML) often goes without a definitive treatment allowing a fibrotic repair paradigm that leads to persistent muscle weakness and long-term disability [2, 3]. The injured muscle can be treated with free muscle transfer [4–6], which is not ideal due to donor site morbidity and continued challenges to functional recovery [7]. There is currently a lack of clinical therapies with the capacity to regenerate a functional volume of muscle tissue on a scale of relevance to clinical extremity trauma.

In recent years, preclinical [8–15] and clinical investigations [16, 17] aimed at VML repair have largely utilized acellular biological scaffolds of various preparations as a therapy that relies on the recruitment of the myriad of cells involved in adult muscle regeneration from the host. Some of these preclinical studies suggest that treatment of VML with acellular biological scaffolds can improve the functional capacity of VML affected skeletal muscle [9, 18, 19]. These observed improvements in functional capacity, however, are correlated with observations of a mixed healing response exhibiting significant fibrous matrix deposition and limited myofiber regeneration at the site of the defect [8, 20]. The specific reason for this muted regenerative response is not completely understood, although it has been observed that very few pax7^+^ cells (i.e. satellite cells) reside beyond the periphery of the VML defect region when treated with devitalized muscle grafts [19], acellular biological scaffolds [8], or cross-linked gelatin hydrogel [21]. This consistent observation indicates that acellular biological scaffolds promote either an insufficient chemotactic signaling gradient to induce satellite cell migration to the middle of the defect or an inhospitable environment to satellite cell survival.

Implantation of autologous minced muscle grafts (~1 mm^3^ pieces of autologous muscle tissue) is a developmental treatment paradigm effective in promoting *de novo* muscle fiber regeneration and functional recovery in small and large animal models of VML [22, 23]. Mincing of the autologous tissue causes the adult fibers to die but delivers viable muscle progenitors and basal lamina required for *de novo* skeletal muscle regeneration [24, 25] and induces a canonical muscle injury response that promotes fiber regeneration [26]. Devitalization and donor cell-labeling experiments indicate that minced grafts deliver muscle progenitors (e.g., satellite cells) that directly contribute to skeletal muscle fiber regeneration [19, 27–29]. A significant shortcoming of the use of autologous muscle tissue is the obligatory donor tissue burden for the repair of clinically large VML defects. To this end, *expansion* of minced grafts, is recognized as a strategy to reduce the tissue sourcing burden limiting the application of minced grafts for the repair of large VML defects [30, 31].

The apparent limitation of each of these approaches (acellular bioscaffold and minced muscle grafts; MG) provides an opportunity for a synthesis of concepts. That is, we envision a treatment paradigm in which the co-delivery of acellular bioscaffold reduces the autologous tissue burden associated with MG by providing a myoconductive microenvironment directly adjacent to the graft material, and the homogeneous distribution of MG throughout the defect space provides a myogenic cell source to critical regions of the defect space, as well as a putative myoinductive environment for infiltrating cells. This is a treatment paradigm that offers a potential off-the-shelf and point-of-care solution for otherwise debilitating VML injuries. Herein, we test the hypothesis of acellular bioscaffold material expansion of autologous minced muscle grafts in a rat model of VML injury.

## Materials & methods

### Animals

This study was conducted in compliance with the Animal Welfare Act, the implementing Animal Welfare Regulations and in accordance with the principles of the Guide for the Care and Use of Laboratory Animals. All animal procedures were approved by the US Army Institute of Surgical Research Institutional Animal Care and Use Committee (A-14-017). 66 male adult Lewis rats of aged 3–4 months were purchased from Harlan Sprague Dawley Inc. (Indianapolis, IN, USA) for use in this study. A subset of Lewis GFP rats (n = 4) were purchased from the Rat Resource Center. Following their use, all animals were euthanized via intracardiac delivery of lethal dose of sodium pentobarbital (Fatal Plus) while under isoflurane anesthesia.

#### Experimental design

This study was designed to test the hypothesis that an acellular biological scaffold expands regenerative outcomes of a 50% by mass VML defect minced graft implantation. Expansion was operationally defined as a significant improvement of indices of muscle fiber regeneration in the expansion product, consisting of a MG dose matched to 50% of the VML defect mass to be co-delivered with an a micronized acellular bioscaffold, versus treatment with a matched dose of minced muscle graft delivered without scaffolding. To test this hypothesis, rats were divided among a sham control group and five experimental groups that each received VML injury to the middle third of the TA muscle on the left hindlimb: (1) no repair, (2) repair with minced muscle grafts at a dosage of 100% of the VML defect mass (100% MG), (3) repair with minced muscle graft at a dosage of 50% of the VML defect mass (50% MG), (4) repair with micronized urinary bladder matrix (100% UBM), and (5) repair with a combination of minced muscle graft and urinary bladder matrix (50% MG + 50% UBM). Rats survived to 2 and 8 weeks post injury before the TA muscles were harvested, weighed (i.e., muscle wet weight), and prepared for histological and molecular analyses. Prior to harvest at the 8-week time-point, the neural-evoked, isometric tetanic strength of the injured and contralateral uninjured TA muscles was assessed *in vivo*.

### VML model

Under anesthesia (1–3% isoflurane) and in sterile conditions, an incision was made through the skin along the lateral aspect of the tibialis anterior (TA) muscle of the left hind limb of adult male Lewis rats. Skin and fascia were reflected from the anterior surface, and a metal spatula position between the TA muscle and underlying extensor digitorum longus (EDL) muscle after blunt dissection. A full thickness defect from the belly of the TA was excised using a 6 mm biopsy punch and then weighed (i.e., defect weight). Wounds were either left unrepaired or repaired with therapies delivered directly to the VML defect region. The wound was closed in layers by suturing (fascia) and stapling (skin). Sham controls received the full operative procedure sans creation of the VML defect via biopsy punch.

### Preparation of minced muscle graft and UBM therapies

Minced grafts were derived from the TA muscle of syngeneic GFP+ transgenic Lewis rats. The excised muscle tissue was minced into ~1mm^3^ pieces. Based on experimental group, minced grafts were either delivered directly to the VML defect region at the specified dosage (50% or 100% of VML defect mass) or manually mixed into a slurry with micronized UBM (MatriStem MicroMatrix, Lot# LP5581-50, ACell Inc., Columbia, MD) prior to implantation. For the delivery of UBM, a dosage of 50% of the VML defect mass was determined to fully fill the defect volume. As such, the UBM alone treatment group (100% UBM) received a full dosage of UBM at 50% of the VML defect mass, while the co-delivery group (50% MG + 50% UBM) received a dosage of UBM at 25% of the VML defect mass (50% of the full UBM dose). Therapies were delivered to the VML defect immediately after preparation (**Table 1**).

**Table 1:** Preparation of regenerative therapies.

	No Repair	100% UBM	50% MG	50% MG 50% UBM	100% MG
MG Delivered [mg]	-	-	42.08 ± 0.65	43.48 ± 0.92	86.71 ± 2.17
UBM Delivered [mg]	-	44.74 ± 1.09	-	21.80 ± 0.42	-

### In vivo functional assessments

The knee and ankle joints were fixed at right angles with the animal in the supine position. The foot was strapped into a pedal coupled with a servomotor controlled force-displacement transducer (Aurora Scientific, Aurora, ON). The anterior crural muscles were stimulated using percutaneous needle electrodes placed around the peroneal nerve. Optimal voltage was determined using a series of twitch and tetanic contractions. Contractile function of the TA muscle was assessed by first severing the distal tendon of the synergist EDL muscle and measuring peak isometric torque as a function of stimulation frequency (400 ms train; 0.1 ms pulse width; 1–10 V; 10–200 Hz;). Previous pilot testing demonstrated that following distal tenotomy the EDL muscle’s contribution to dorsiflexor torque with the ankle 90° is negligible [22].

### Transcriptional analysis

Harvested TA muscles were portioned into thirds using a straight razor blade. The middle third, which visibly contained the defect region, was further separated into a proximal and distal half at the midline of the defect. A cross-sectional piece (~50 mg) of the most proximal aspect of the distal piece the distal was used for transcriptional analysis. The proximal half of the defect was processed for histology (see below). A portion of the defect region of the VML injury was collected at 2 weeks post-injury and immediately snap frozen in liquid nitrogen. RNA was isolated from the homogenized tissue using the TRIzol method. RNA samples were transcribed into cDNA and used for array analysis in the RT^2^Profiler PCR array for innate and adaptive immune responses (Catalog # PARN-052Z, SABiosciences Corp., Frederick, MD, USA) per the manufacturer's instructions. The raw fluorescence data was processed using LinRegPCR (v12.11; http://www.hartfaalcentrum.nl) with Actb, B2m, Hprt1, Ldha, and Rplp1 serving as the endogenous controls through geometric averaging [32, 33]. Expression (n = 3–4 per group) of each target gene was calculated relative to samples from the no repair group.

### Histological & immunofluorescence analysis

The proximal half of the TA muscle belly (inclusive of the defect region) was embedded in a talcum-based gel, frozen in 2-methylbutane, and supercooled in liquid nitrogen. Cryosections (8 μm) were prepared and stained using standard protocols for hematoxylin & eosin and Masson’s trichrome. For immunofluorescence, sections were probed for laminin (1:100; catalog ab34360; Abcam) and either GFP (1:100; catalog ab6673; Abcam), CD68 (1:50; MCA341A488; BioRad), or CD3 (1:50, catalog ab185763; Abcam). Positive staining was detected with fluorescent secondary antibodies (1:200; catalog A11055, A21202, and A21207; Invitrogen). Brightfield and fluorescent images were acquired with a Zeiss Axio Scan.Z1 and stitched into a composite image depicting the cross section of the TA muscle belly. Qualitative assessments of morphology and composition were made by observing three sections from 5 muscles per group. Quantitative Analyses were performed on the indexed image values after global thresholding and segmentation in MATLAB (Mathworks, Natick, MA, USA). Defect regions were defined based on visual inspection and qualitative assessments of myofiber size and organization. Remaining muscle mass was defined as the remainder of the section not included in the defect region.

### Statistical analysis

Data is reported as the mean ± SEM with statistically significant differences defined as p < 0.05 by t-tests and analysis of variance with Holm-Sidak post-hoc tests for multiple comparisons as appropriate. Sample sizes for gene expression, quantitative immunohistochemistry, and *in vivo* neuromuscular strength assessments are n = 3–4, n = 5–6, and n = 5–6 per group, respectively.

## Results

### Body and muscle weights

There were no differences between experimental groups with respect to body weight at either of the 2 and 8 week study endpoints (**Table 2**). The mean wet weight of the VML defect was similar across all groups. The mean wet weight of the EDL or TA muscles from the unaffected limbs did not vary significantly between groups at either of the study endpoints. At 2 weeks post-injury TA muscle wet weight in VML affected limbs were reduced relative to their matched contralateral limbs. Among the VML affected limbs, the TA wet weights for the 100% UBM group was significantly higher than all other treatment groups at 2 weeks post-injury. At 8 weeks post-injury, TA wet weights were similar between treatment groups.

**Table 2:** Gross anatomy & muscle weights.

			No Repair			100% UBM			50% MG			50% MG 50% UBM			100% MG		
Defect Weight [mg]		2 Weeks	82.3	±	1.9	86.0	±	3.8	84.9	±	1.8	86.9	±	2.9	85.6	±	3.1
		8 Weeks	88.4	±	4.2	87.9	±	1.2	83.3	±	2.1	86.7	±	1.5	87.9	±	3.1
Endpoint Body Weight [g]	†	2 Weeks	349	±	2.8	359	±	3.1	350	±	4.2	347	±	11.2	344	±	6.4
		8 Weeks	447	±	8.1	441	±	11.1	453	±	9.7	455	±	11.8	458	±	5.0
TA Weight [g]		2 Weeks	0.430	±	0.033	0.669	±	0.038	0.478	±	0.029	0.483	±	0.019	0.507	±	0.029
		8 Weeks	0.690	±	0.033	0.769	±	0.050	0.814	±	0.053	0.810	±	0.040	0.774	±	0.024
EDL Weight [g]		2 Weeks	0.113	±	0.007	0.120	±	0.003	0.127	±	0.008	0.126	±	0.012	0.109	±	0.005
		8 Weeks	0.182	±	0.012	0.209	±	0.017	0.184	±	0.016	0.210	±	0.016	0.182	±	0.016

† Significant main effect between timepoints (p<0.05).

### Qualitative histological observations

**Fig 1:**
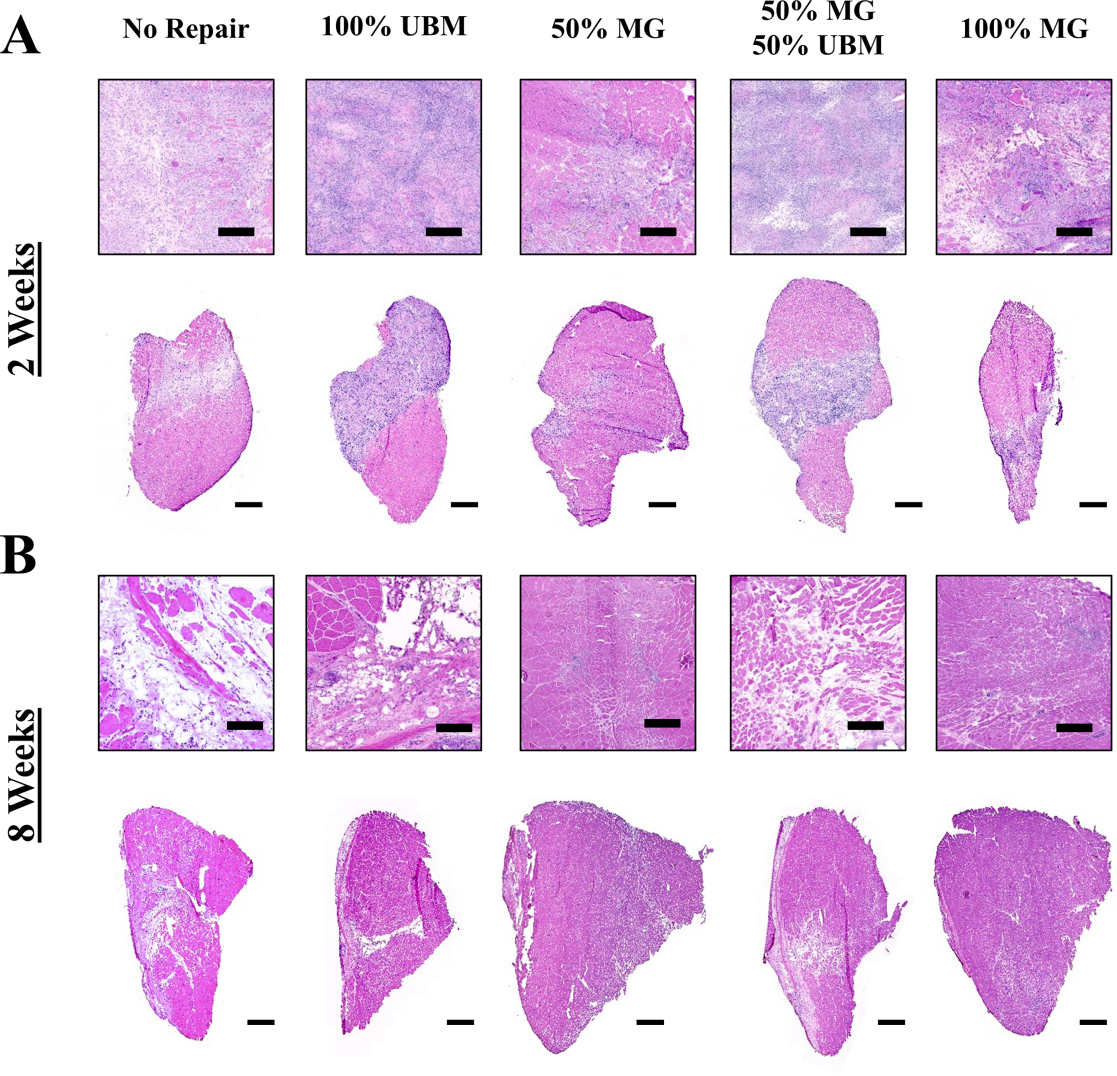
Histological assessment of skeletal muscle wound healing. Hematoxylin & Eosin staining of VML affected TA muscle at A) 2 weeks and B) 8 weeks post-injury from the 50% and 100% MG treatment groups exhibit similar results at 8 weeks post injury. The expansion test group (50% MG + 50% UBM) presents greater muscle fiber presence relative to UBM alone, but exhibits greater deposition of non-contractile tissue in the defect space relative to the MG alone groups (50% MG, 100% MG). Scale bars represent 200 and 1000 microns at high and low magnification, respectively.

Histologically, the unrepaired control group exhibited substantial cellular infiltration of the defect space at 2 weeks post-injury. This observation is paired with a terminal wound healing outcome consisting of considerable non-contractile tissue mixed with sparsely distributed nascent myofibers (Fig 1). The 100% UBM treatment group likewise exhibited significant cellular infiltration of the defect region at 2 weeks, which did not result in robust myofiber regeneration by 8 weeks. The 50% MG and 100% MG groups showed similar levels of cell infiltration and evidence of minced graft degeneration at 2 weeks post-injury. The 8 week healing outcomes for these groups were also similar, exhibiting evidence of skeletal muscle regeneration within the defect region in the form of disorganized myofibers of noticeably reduced cross-sectional area relative to the surrounding musculature. The 50% MG + 50% UBM treatment group showed robust cellular infiltration of the VML defect region at 2 weeks post-injury. The 8 week healing outcome of this group was mixed, exhibiting the presence of nascent myofibers interspersed with non-contractile tissue deposition.

### GFP^+^ muscle fiber regeneration

**Fig 2:**
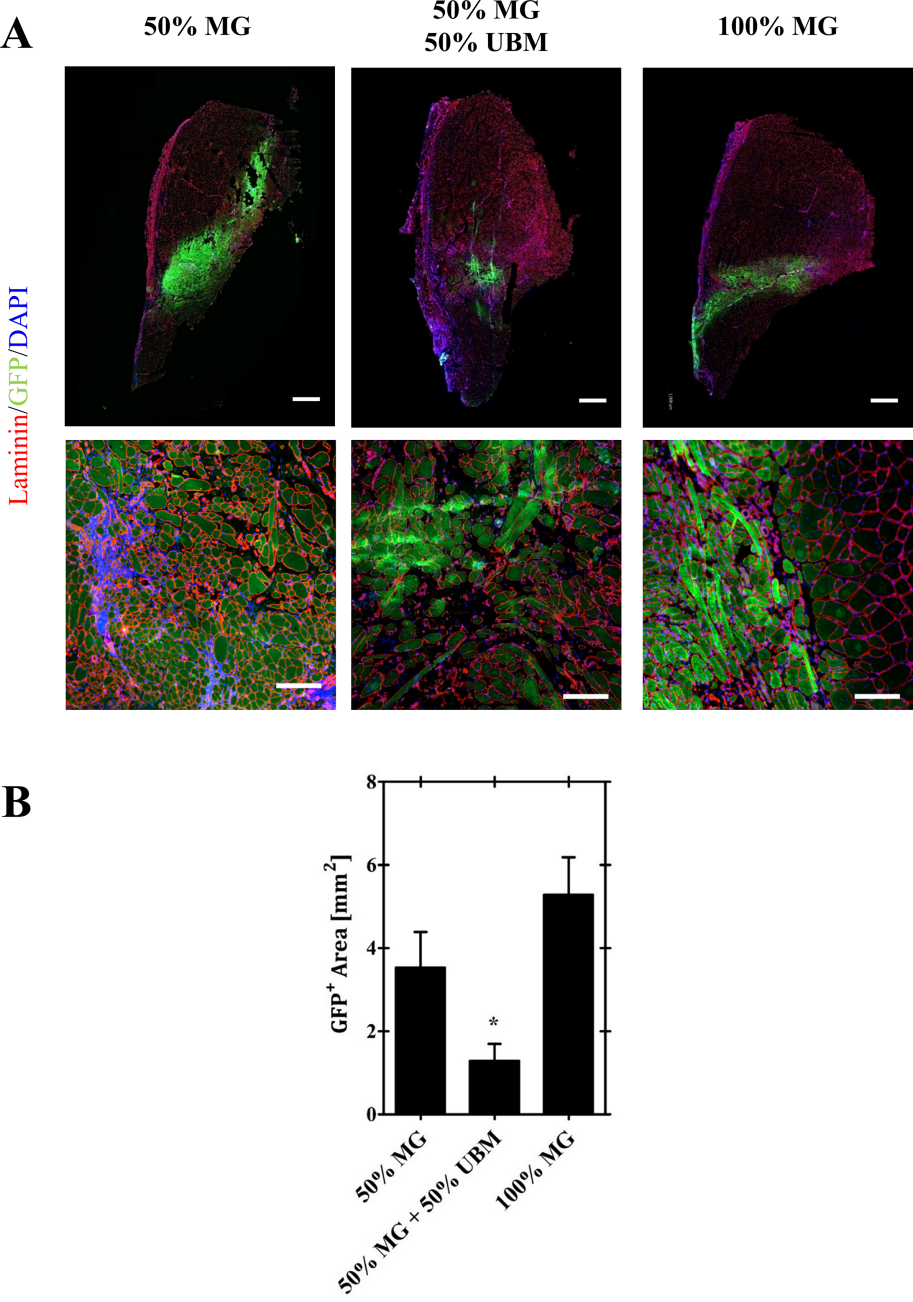
Minced graft contribution to regeneration. A) Immunofluorescence staining (above) of GFP+ tissue within the defect region of VML affect TA muscles reveals reconstitution of defect space to include *de novo* myofibers and collagenous tissue by minced graft derived cells. B) GFP+ area is similar between 50% MG and 100% MG groups, but decreased in the expansion group (50% MG + UBM). Quantitative analysis of total GFP+ area was performed on 6 muscles per group. *Indicates a statistically significant difference between groups (p<0.05).

GFP was detected in the defect region of the 50% MG, 50% MG + 50% UBM, and 100% MG treatment groups out to 8 weeks post-injury indicating the viable engraftment of the graft derived cells and subsequent involvement in myofiber regeneration (Fig 2). GFP^+^ staining indicated that minced graft-derived muscle progenitors significantly contributed to the regeneration of muscle fibers within the defect region and the graft- derived cells did not abundantly migrate into the remaining musculature surrounding the defect area. Quantitative assessment of total GFP^+^ staining indicated that 50% MG and 100% MG treatments result in similar muscle fiber regeneration within the defect region (p = 0.190). 50% MG + 50% UBM treatment conversely resulted in significantly reduced graft derived tissue deposition relative to both the 50% MG (-63.6%, p = 0.038) and 100% MG (-75.6% deficit, p = 0.002) groups.

### In vivo neuromuscular function

**Fig 3:**
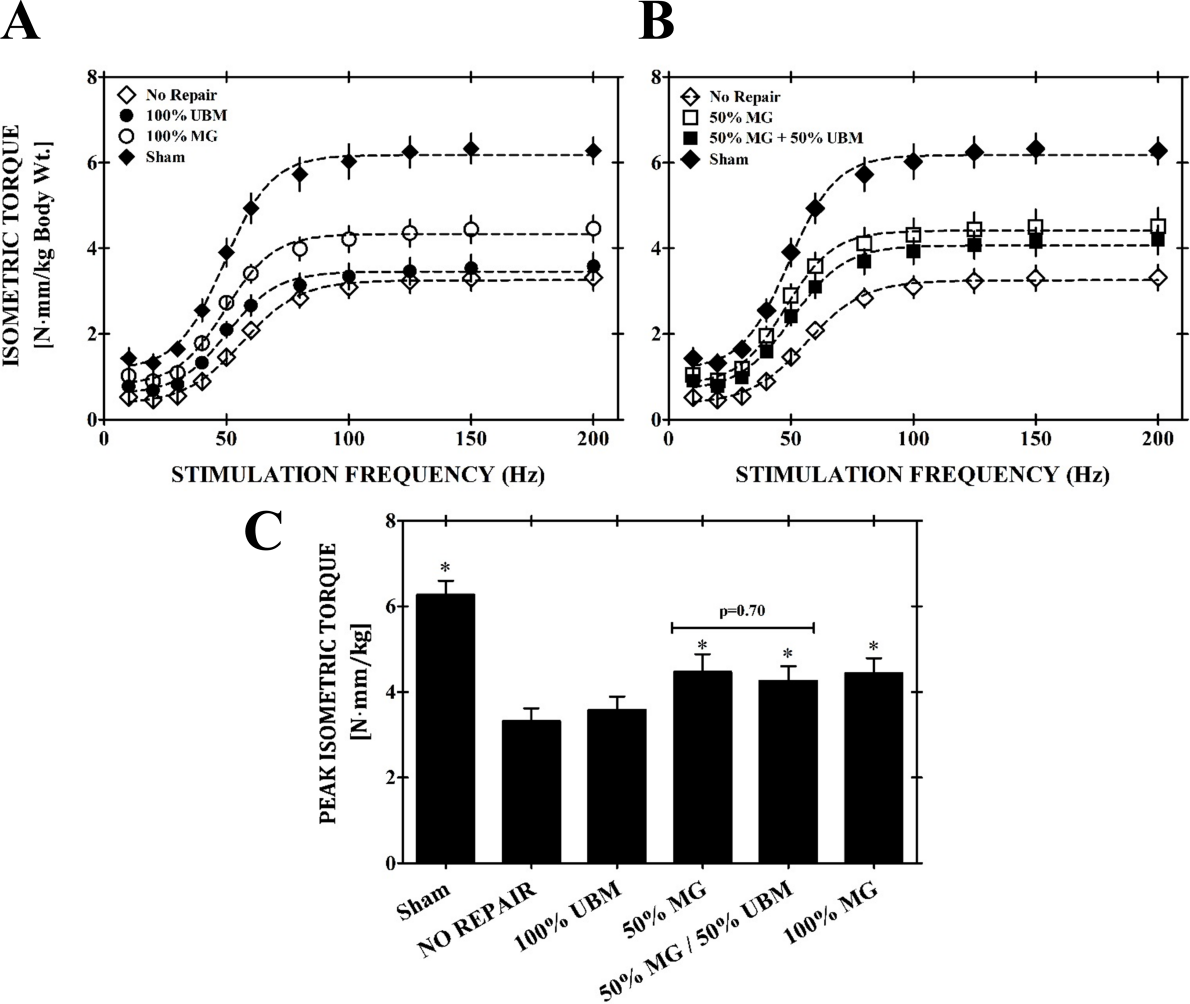
Neuromuscular functional capacity assessments. In vivo TA muscle neuromuscular strength was assessed at 8 weeks post injury for A) No Repair, 100%UBM, 100% MG, Sham-operated control groups and B) 50% and 50% MG + 50% UBM experimental groups. No differences were observed between the expansion product (50% MG + 50% UBM) and either of the minced graft treated groups (50% MG, 100% MG). *Indicates a statistically significant increase in peak isometric torque for the groups denoted relative to the no repair group (p<0.05). 50% MG, 50% MG + 50% UBM, and 100% MG treatment groups all exhibited significantly higher isometric torque for all frequencies above 50 Hz relative to the No Repair control group.

At 8 weeks post injury, peak isometric tetanic torque was significantly lower in all VML affected experimental groups relative to sham controls. Mean peak isometric torque production of unrepaired, VML affected limbs was 3.32 ± 0.30 N·mm per kilogram of body weight, representing a 47.1 ± 4.4% torque deficit compared with limbs that received a sham surgery (Fig 3**).** Within the treatment groups, isometric torque generation was significantly increased at stimulation frequencies greater than 50 Hz in the 100% MG, 50% MG, and 50% MG + 50% UBM groups (150 Hz; 4.44 ± 0.35, 4.47 ± 0.41, & 4.26 ± 0.34 Nmm/kg, respectively). In terms of mean peak isometric torque, this corresponds to a 34.7%, 33.7%, and 28.2% improvement relative to the no repair group for the 50% MG, 100% MG, & 50% MG + 50% UBM groups, respectively. Peak isometric torque generation for the 100% UBM (3.58 ± 0.32 N·mm/kg, 150Hz) was not significantly different from the VML unrepaired group at any stimulation frequency.

### Assessment of immune cell infiltration

**Fig 4:**
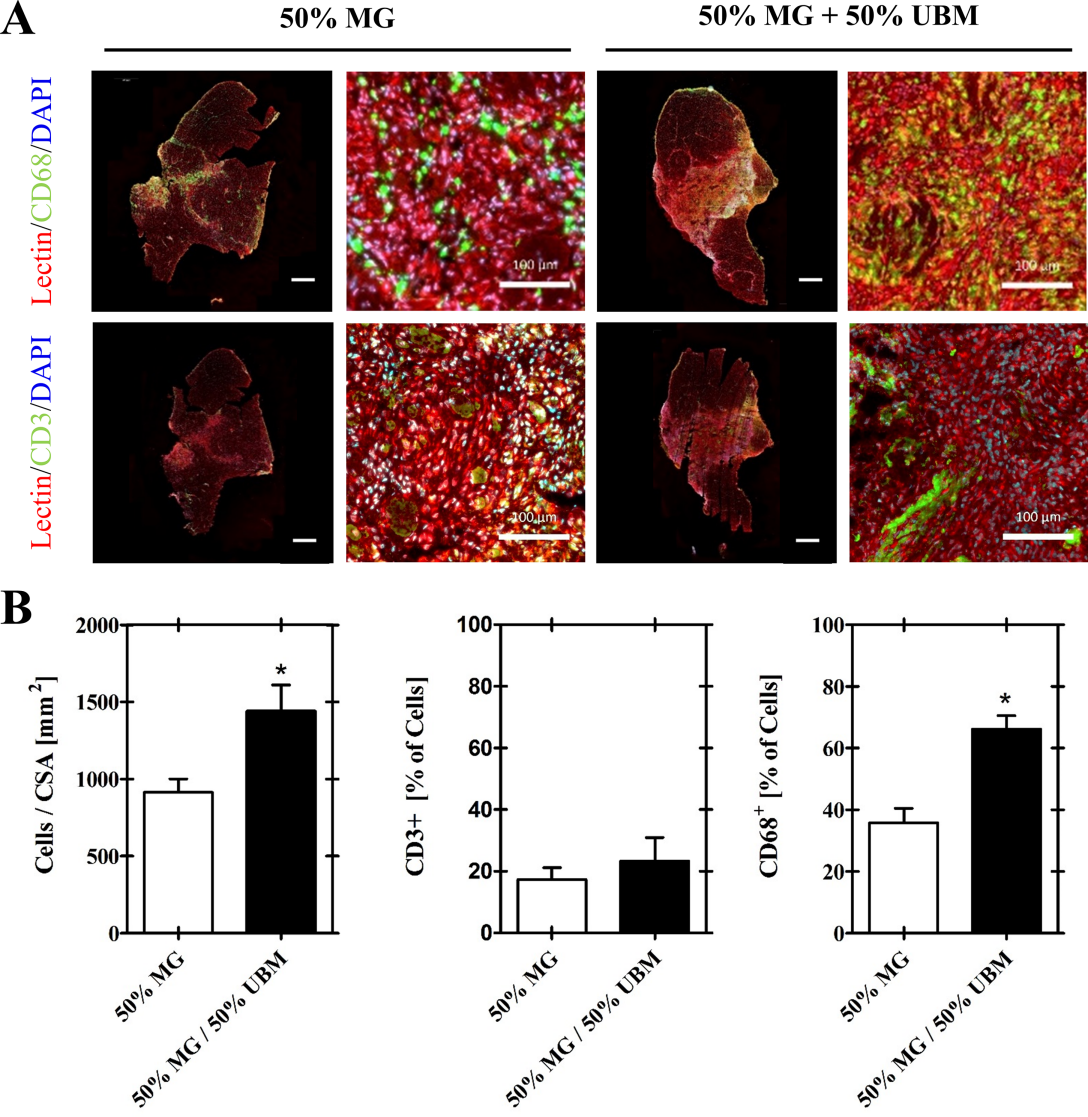
Immunofluorescent analysis of immune cell infiltrate. A) Immunofluorescence staining depicts immune cell infiltration of the VML defect space with CD3^+^ T lymphocytes and CD68^+^ macrophages at two weeks post injury in both the expansion product group (50% MG + 50% UBM) and its dosage matched minced graft control (50% MG). B) Quantitative analysis reveals the overall cellularity was significantly increased in the expansion product group relative to the 50% MG control. Furthermore, the increase in cellularity was primarily driven by the CD68+ population as CD68 expression was significantly more robust in the expansion group than the 50% MG control. Scale bars represent 100 and 1000 microns at high and low magnification, respectively. * Indicates a statistically significant difference between groups (p<0.05). Representative images intensities have been enhanced for illustrative purposes.

In light of the clear role of innate and adaptive immune responses to skeletal muscle regeneration and scaffold-mediated tissue remodeling, we questioned whether the diminution of muscle fiber regeneration observed in the expansion product (Fig 2) was due to exacerbated or dysregulated inflammation. Quantitation of the cellular infiltrate at 2 weeks post-injury indicated cellularity (i.e. nuclei counts, DAPI) was significantly higher (p<0.05) in the 50% MG + 50% UBM treatment group relative to the 50% MG treatment group (Fig 4). CD3^+^ T-cell populations in the 50% MG + 50% UBM and the 50% MG treatment groups were similar (p = 0.31). However, CD68^+^ macrophage density was significantly higher in the 50% MG + 50% UBM treatment group relative to the 50% MG treatment group.

### Transcriptional analysis

**Fig 5:**
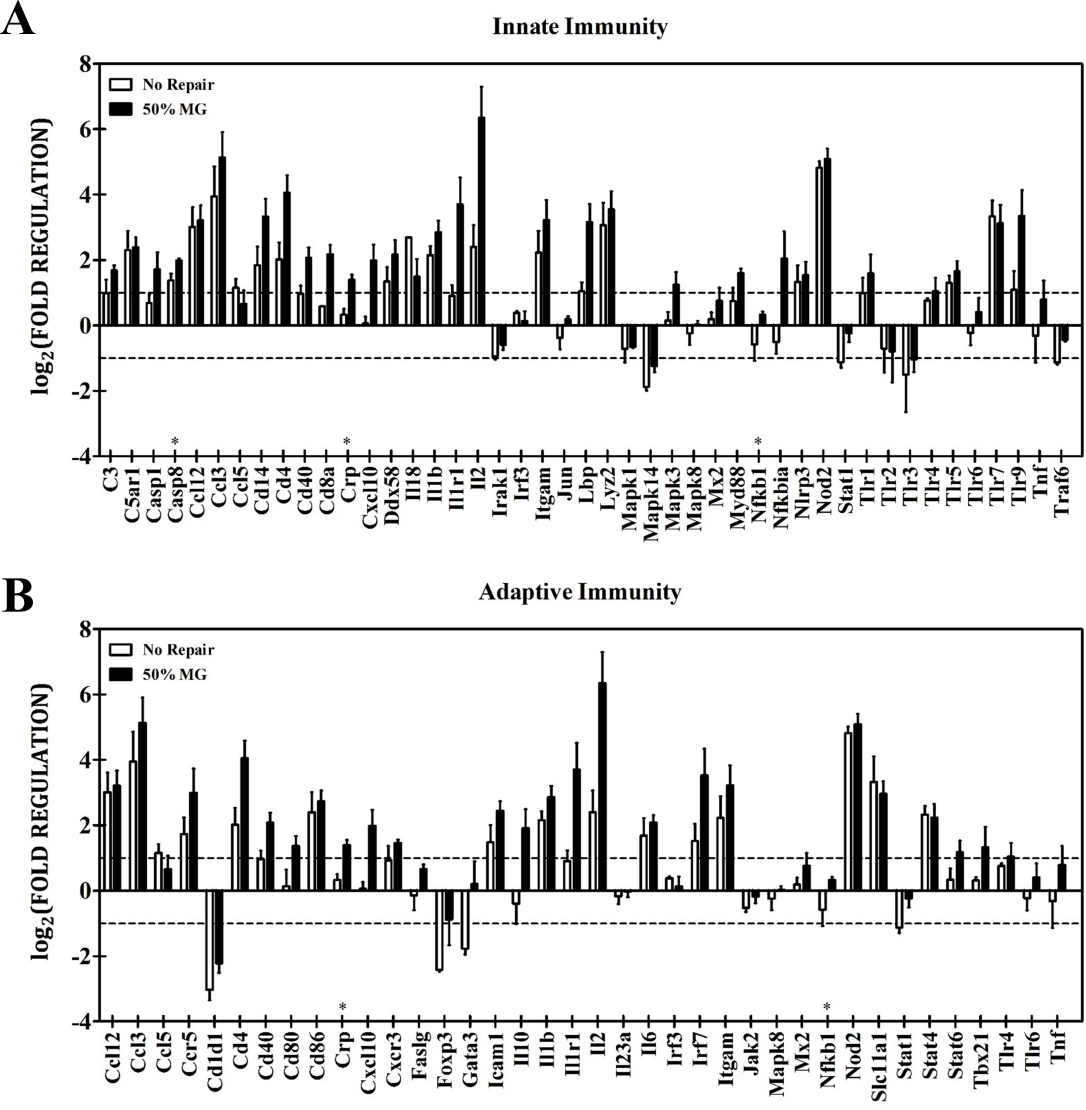
transcriptional analysis of immune response related signaling in VML injury condition with and without minced graft intervention. Bar graphs depict A) innate and B) adaptive immune response related gene expression at two weeks post injury in VML affected limbs in the untreated and 50% MG treated conditions. In general, immune related transcription is meaningfully upregulated (>2 fold change relative to unaffected contralateral limbs) in the untreated condition. Treatment of the wound with 50% MG overwhelmingly does not significantly alter the expression of these subsets of genes in either directions. * Indicates a statistically significant difference between groups (p<0.05).

**Fig 6:**
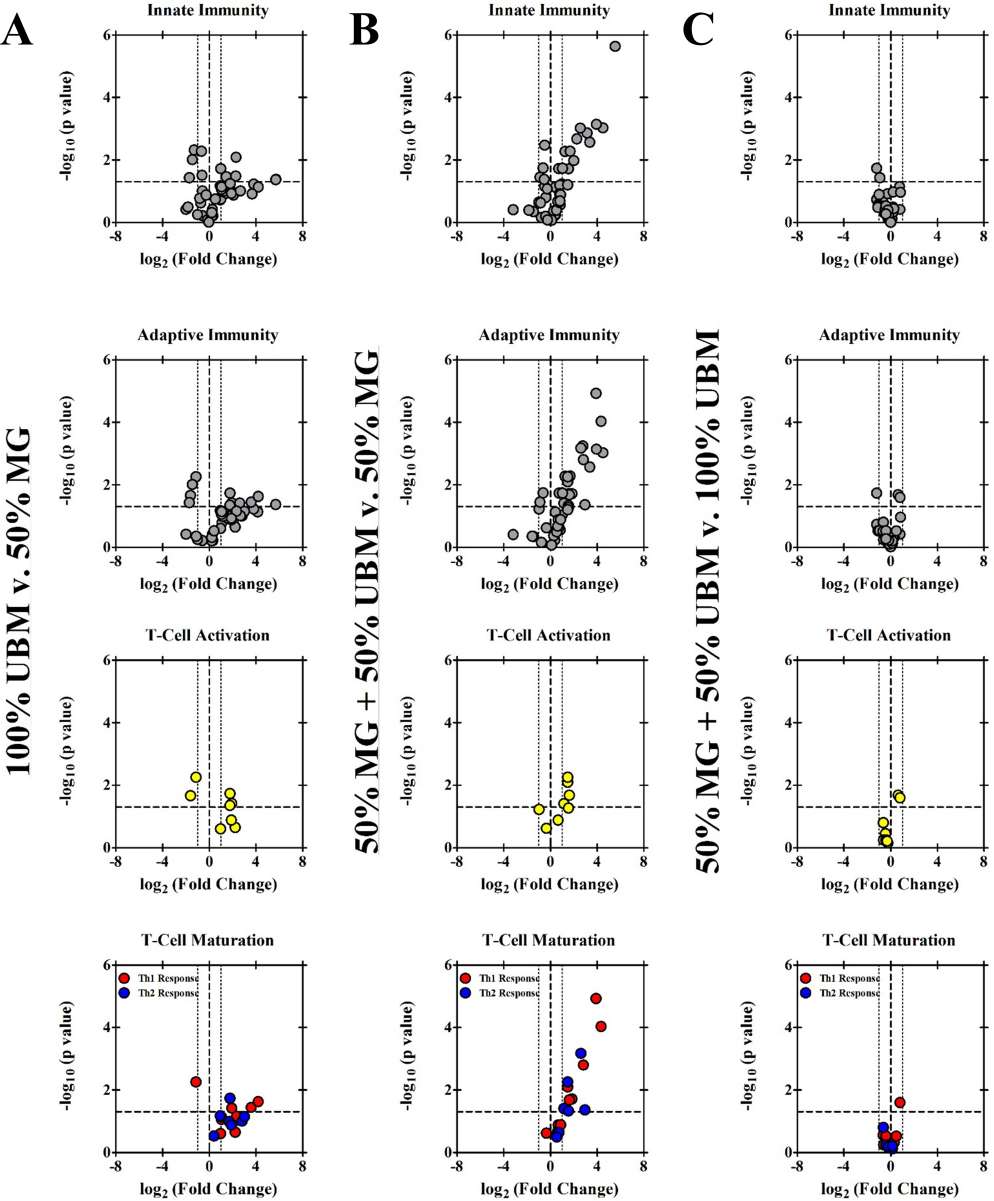
Transcriptional analysis of immune response related signaling between therapeutic groups. Volcano plots depict the differential expression of several immune response related genes for A) 100% UBM vs. 50% MG, B) 50% MG + 50% UBM vs. 50% MG, and C) 50% MG + 50% UBM vs. 100% UBM. Plots are presented as gene panels related to the innate immunity, adaptive immunity, T-Cell activation, and T-Cell Maturation in descending rows starting from the top row. Thick dashed lines mark thresholds for statistical significance on the y-axis (p = 0.05), and the transition point between upregulation and downregulation on the x-axis (fold change = 1). Thinner dashed lines represent a general threshold for biological meaningfulness: fold change = 2 for up-regulated genes and fold change = 0.5 for down-regulated genes.

RT-qPCR array analysis was also performed to identify differential transcriptional levels of several key genes related to the innate and adaptive immune responses at two weeks post-injury (Table 3). At this time, 32 of 84 genes related to innate and adaptive immune responses presented a greater than two-fold change in non-repaired VML injured muscle normalized to uninjured control muscle, indicating a prolonged active immune response at this time post-injury. Gene expression was grossly similar between these unrepaired injured muscles and VML affected limbs treated with 50% MG- a total of 3 genes (*Casp8*, *Crp*, *Traf6*) of the 75 assayed presented statistically different expression (Fig 5), and of the three *Crp* showed the greatest differential expression with a fold change of 2.11. In comparison to 50% MG, limbs treated with 100% UBM or 50% MG + 50% UBM presented significant upregulation of immune related transcriptional activity. Relative to the 50% MG treatment group, the 100% UBM and 50% MG + 50% UBM treatment groups showed significantly increased transcription of genes related to the promotion of the inflammatory response, production of pattern recognition receptors, broad cytokine production, T-cell activation, and the maturation of both type-1 and type-2 T-helper cells (Fig 6). For the 50% MG + 50% UBM group, genes related to the inflammatory response that were significantly upregulated relative to the 50% MG group included *C3* (46.8 FC), *Ccl5* (15.5 FC), *Il1b* (3.2 FC), and *Il6* (2.8 FC). Additionally, the 50% MG + 50% UBM group exhibited significant upregulation of the following genes broadly related to cytokine production: *Ccl12* (22.8 FC), *Cxcl10* (3.0 FC), and *Il10* (6.2 FC). Additional genes related to the downstream activation and maturation of T-cell populations that were upregulated in the 50% MG UBM group relative to the 50% MG group included *Ccr5* (3.6 FC), *Cd80* (2.8 FC), *Cd86* (2.2 FC), *Cxcr3* (15.0 FC), *Gata3* (2.9 FC), Stat4 (20.3 FC), and *Tbx21* (7.1 FC). Of this set of genes, *Ccr5*, *Cxcr3*, *Stat4*, *Tbx21* are more closely associated with a Th1 phenotype as opposed to a Th2 phenotype (*Cd86*, *Gata3*) or T-cell activation processes (*Cd80*). On account of the 50% MG + 50% UBM group presenting less variability than 100% UBM, a greater number of genes were significantly differentially expressed compared to 50% MG only (50% MG + 50% UBM vs. 100% UBM: 32 vs. 22 genes were statistically differentially expressed). However, only four of the 75 genes (analyzed were significantly upregulated or downregulated between the 50% MG + 50% UBM and 100%UBM groups (*Cd1d1*, *Il2*, *Il23a*, and *Lyz2*) and none of those four exhibited a relative change in expression greater than the -2.3 FC exhibited by *Il2* in the expansion group relative to the UBM control, indicating a similarly exacerbated inmmune response between the treatment groups involving UBM-repair **(**Table 3**)**.

**Table 3:** Transcriptional analysis of immune response.

	50% MG			100% UBM				50% MG 50% UBM			
Actb	1.90	±	0.33	2.57	±	0.25		2.08	±	0.06	
B2m	0.99	±	0.19	2.46	±	0.26	*	2.47	±	0.06	*
C3	1.63	±	0.15	85.83	±	22.56	*	76.10	±	1.50	*
C5ar1	1.06	±	0.20	3.16	±	0.70		2.08	±	0.25	
Casp1	2.04	±	0.73	3.91	±	0.72		2.76	±	0.21	
Casp8	1.53	±	0.04	3.02	±	0.32	*	2.51	±	0.20	*
Ccl12	1.15	±	0.35	60.01	±	15.96	*	26.30	±	2.31	*
Ccl3	2.28	±	1.31	1.50	±	0.31		1.35	±	0.27	
Ccl5	0.71	±	0.19	12.66	±	3.87		10.92	±	0.87	*
Ccr4	7.26	±	1.52	50.59	±	11.71		56.53	±	9.25	*
Ccr5	2.38	±	1.14	13.47	±	2.87		8.68	±	0.43	*
Ccr6	0.96	±	0.13	11.48	±	2.33	*	10.75	±	2.37	*
Cd14	2.82	±	1.04	5.59	±	1.04		3.51	±	0.32	
Cd1d1	1.75	±	0.26	0.57	±	0.04	*	0.90	±	0.06	†
Cd4	4.07	±	1.53	5.03	±	0.52		5.29	±	0.68	
Cd40	2.16	±	0.43	7.60	±	1.89		5.19	±	0.14	*
Cd40lg	1.27	±	0.38	17.88	±	3.67		13.28	±	0.78	*
Cd80	2.33	±	0.45	8.89	±	1.67	*	6.51	±	0.52	*
Cd86	1.26	±	0.26	4.38	±	0.63	*	2.83	±	0.34	*
Crp	2.11	±	0.19	0.65	±	0.35	*	1.13	±	0.17	*
Cxcl10	3.80	±	1.22	16.11	±	4.95		11.23	±	1.05	*
Cxcr3	1.44	±	0.09	17.34	±	4.10	*	21.69	±	0.61	*
Ddx58	1.77	±	0.51	1.07	±	0.07		0.88	±	0.02	
Faslg	1.74	±	0.16	10.71	±	2.35	*	11.84	±	0.82	*
Foxp3	2.91	±	1.01	6.16	±	1.25		5.80	±	0.40	
Gata3	3.93	±	1.99	12.90	±	2.86		11.40	±	0.80	*
Hprt1	1.32	±	0.40	0.83	±	0.06		0.70	±	0.04	
Icam1	1.95	±	0.36	6.65	±	1.26	*	5.69	±	1.01	
Ifnar1	1.26	±	0.00	3.46	±	1.14		1.62	±	0.18	
Ifng	0.96	±	0.33	4.49	±	1.84		2.97	±	0.30	*
Ifngr1	1.13	±	0.25	1.83	±	0.15		1.42	±	0.10	
Il10	4.92	±	2.01	39.45	±	10.95		30.29	±	0.80	*
Il1a	0.40	±	0.09	7.36	±	2.27		3.54	±	0.31	*
Il1b	1.63	±	0.36	10.33	±	3.11		5.28	±	0.40	*
Il1r1	6.94	±	4.36	3.48	±	0.94		2.55	±	0.16	
Il2	15.34	±	11.68	3.86	±	0.43		1.69	±	0.21	†
Il23a	1.10	±	0.10	0.50	±	0.06	*	0.88	±	0.09	†
Il6	1.31	±	0.18	4.86	±	1.44		3.67	±	0.30	*
Irak1	1.28	±	0.10	0.86	±	0.13		0.92	±	0.06	
Irf3	0.84	±	0.15	1.01	±	0.06		1.02	±	0.06	
Irf7	4.02	±	2.55	1.85	±	0.31		1.36	±	0.25	
Itgam	1.99	±	0.87	5.59	±	1.22		3.59	±	0.60	
Jak2	1.27	±	0.13	2.80	±	0.52		2.13	±	0.13	*
Jun	1.48	±	0.09	0.60	±	0.12	*	1.02	±	0.09	*
Lbp	4.34	±	1.67	3.92	±	0.47		3.26	±	0.51	
Ldha	0.82	±	0.06	0.34	±	0.07	*	0.49	±	0.06	
Lyz2	1.40	±	0.53	3.79	±	0.46	*	2.00	±	0.06	†
Mapk1	1.05	±	0.02	0.66	±	0.06	*	0.75	±	0.03	*
Mapk14	1.55	±	0.16	1.01	±	0.05	*	1.19	±	0.05	
Mapk3	2.14	±	0.54	1.22	±	0.14		1.20	±	0.13	
Mapk8	1.21	±	0.08	0.44	±	0.13	*	0.79	±	0.04	*
Mx2	1.49	±	0.39	1.73	±	0.19		1.60	±	0.23	
Myd88	1.80	±	0.15	4.06	±	0.71		2.89	±	0.35	
Nfkb1	1.88	±	0.10	2.18	±	0.28		2.51	±	0.19	
Nfkbia	5.86	±	3.77	1.66	±	0.22		1.64	±	0.14	
Nlrp3	1.15	±	0.31	5.62	±	0.74	*	4.62	±	0.54	*
Nod2	1.21	±	0.24	2.31	±	0.30		2.02	±	0.37	
Rorc	0.55	±	0.10	0.74	±	0.11		0.76	±	0.17	
Rplp1	0.93	±	0.12	0.98	±	0.04		0.87	±	0.02	
Slc11a1	0.78	±	0.19	1.52	±	0.40		1.23	±	0.06	
Stat1	1.87	±	0.27	4.16	±	0.82		3.85	±	0.32	*
Stat3	1.20	±	0.17	1.26	±	0.07		1.30	±	0.10	
Stat4	0.94	±	0.25	17.21	±	3.69	*	18.99	±	0.90	*
Stat6	1.79	±	0.40	2.38	±	0.22		2.62	±	0.28	
Tbx21	2.02	±	0.89	10.18	±	2.50		14.30	±	0.97	*
Tlr1	1.52	±	0.62	19.44	±	7.07		9.03	±	0.34	*
Tlr2	0.94	±	0.37	4.52	±	0.85	*	4.56	±	0.19	*
Tlr3	1.37	±	0.26	3.31	±	0.68		2.40	±	0.20	
Tlr4	1.21	±	0.33	2.50	±	0.37		1.88	±	0.17	
Tlr5	1.27	±	0.25	1.83	±	0.19		2.19	±	0.17	
Tlr6	1.54	±	0.44	3.22	±	0.43		2.84	±	0.33	
Tlr7	0.86	±	0.33	3.04	±	0.60		1.53	±	0.14	
Tlr9	4.80	±	2.83	4.71	±	0.52		4.04	±	0.22	
Tnf	2.14	±	0.87	7.96	±	2.26		5.97	±	0.86	
Traf6	1.62	±	0.05	1.36	±	0.10		1.35	±	0.08	

Note: All data are fold changes relative to the No Repair group (means ± SEM)

* Indicates differential expression relative to the 50% MG treatment group (p<0.05)

† Indicates differential expression relative to the UBM treatment group (p<0.05)

## Discussion

VML injuries cause a permanent loss of skeletal muscle tissue and chronic strength deficits that are not responsive to physical rehabilitation and for which effective regenerative therapies are not clinically mature. Herein, we sought to build off of prior work demonstrating that autologous minced muscle grafts bear the resident elements necessary to promote *de novo* muscle fiber regeneration in whole muscle ablation [26, 34, 35] and VML (partial ablation) [19, 22, 23] models, by taking a combinatorial therapeutic approach to expand the regenerative potential with co-delivery of micronized acellular biological scaffold. Our primary goal was to demonstrate enhanced muscle tissue regeneration mediated by autologous minced grafts through an obligatory myoconductive functionality of the implanted scaffold material. Unfortunately, minced graft mediated skeletal muscle regeneration was actually damped 8 weeks post-injury in the graft-material expansion product, indicating that the UBM micronized scaffold was not conducive to regenerative expansion of minced grafts *in vivo*. Previously, we unsuccessfully attempted to expand autologous minced grafts in a collagen hydrogel, wherein the magnitude of skeletal muscle fiber regeneration following 50% MG repair was approximately half of that observed with 100% MG repair, despite co-delivery of the collagen hydrogel [23]. We rationalized that suspension of minced grafts in the micronized UBM used in this study would yield better results due to the inclusion of laminin, an important substrate for satellite cell activity [36], and the putative inclusion of ECM-bound growth factors, but this modification was to no avail. Two plausible explanations for this suboptimal outcome include the lack of a bulk mechanical structure of the micronized UBM and the inadequacy of the UBM material itself to promote the proper spatiotemporal migration of cellular phenotypes to the defect region due to the complex combination of adhesion sites and signaling molecules which comprise the scaffold. If the latter explanation is in fact true, perhaps a more tightly engineered biomaterial that mimics only the essential components of the ECM necessary to preferentially promote stem cell migration into the defect rather than a more inflammatory infiltrate may ultimately prove useful in the treatment of VML.

Innate and adaptive immune responses play a crucial role in determining skeletal muscle regenerative outcomes. Both pro- and anti-inflammatory cytokine signaling interact with myogenic regulatory pathways, in which an appropriate temporal conversion from type-1 (M1/Th1) to type-2 (M2/Th2) wound healing phenotypes is required for optimal regeneration. Based on evidence from the literature that prolonged inflammation during muscle regeneration can lead to a fibrotic response [37, 38] rather than myogenic repair [39], we questioned whether the inflammatory response within the 50% MG + 50% UBM repaired muscle was exacerbated in a manner that plausibly led to the observed deterioration of skeletal muscle degeneration, when compared to 50% MG alone. In support, general cellular infiltration and specifically CD68+ macrophage infiltration was increased in the expansion group. Moreover, the 50% MG + 50% UBM group presented a gene expression profile representative of an heightened inflammatory response with genes related to activation of the complement system (*C3*), chemotactic homing of T cells and monocytes (*Ccl5* and *Ccl12*), and promotion of a type-1 wound healing phenotype (*Ccr5*, *Cd80*, *Cxcr3*, *Stat4*, *Tbx21*) being amongst the most highly differentially expressed genes among those studied relative to 50% MG alone. While most of these highly regulated genes could generally be characterized as proinflammatory, it is worthwhile to note that *Il10*, an anti-inflammatory cytokine[40], is also upregulated (6.2 FC) in the 50% MG + 50% UBM relative to the 50% MG group. Given the prominent role of the Il10 in anti-inflammatory or immune response resolving responses[41], one possibility for the up-regulation of *Il10* is to attempt to damp the prolonged pro-inflammatory immune response in the tissue.

Of significant interest amongst the highly regulated proinflammatory genes are *Ccl5* and one of its receptors of greatest affinity, *Ccr5*. These genes are of interest in this context due to the fact that their upregulation is associated with chronic inflammation and fibrogenic diseases such as scleroderma [42], pulmonary sarcoidosis [43], and chronic liver disease [44]. Furthermore, *Ccl5* may also play a significant role in acute transplant rejection via signaling through its *Ccr1* receptor [45, 46]. These findings when correlated with our histological observations of a mixed fibrotic/regenerative response in the expansion product group support the working hypothesis that the immune response to the UBM is damping the regenerative response of the minced grafts in the co-delivered expansion group rather than supporting regeneration as intended. Further research into the mechanisms by which acellular bioscaffolds may promote a fibrotic wound healing phenotype and how such an outcome may be mitigated are warranted.

The current immunological observations are particularly interesting given recent reports on the immunomodulatory and pro-regenerative properties of acellular bioscaffolds for ECM repair [14, 15, 47]. The overarching thesis of this body of literature is that acellular bioscaffolds of various origins and preparations promote a microenvironment in which a constructive type II immune response (M2/Th2) at the site of injury prevails over the proinflammatory type I immune response (M1/Th1), a shift in polarization which has been shown to be critical to endogenous skeletal muscle regeneration of recoverable injuries [48]. The results of the current study agree with Dziki et al. [15] and Sadtler et al. [49] with respect to the role of acellular bioscaffolds in promoting a robust cellular infiltration response during the early stages of skeletal muscle wound healing and the cellular response being comprised primarily of myeloid cells. Moreover, the mixed response at two weeks may reflect an intermediate phase between the immune phenotype shift that occurred between 1 and 3 weeks post-injury follow acellular scaffold repair of mouse quadriceps VML [49]. However, the current findings of a mixed M1/M2 response 14 days post-injury, suggests that the early conversion from a mixed M1/M2 response at 3 days post injury to a predominately M2 driven response by 7 days post-injury observed previously may not be sustained [15]. In any case, there is no clear association between phenotypic changes in the immune response and functional skeletal muscle regeneration. In fact, the histological data presented herein appear fairly similar to that reported in Dziki et al. [15], which did not report functional outcomes of regeneration. And, the functional outcomes reported by Sadtler et al., were not different between non-repaired and ECM-repaired VML injured quadriceps muscles in wild-type mice [49]. Each of these studies offer very different interpretations of the capacity of implanted acellular biological materials to inform *de novo* skeletal muscle fiber regeneration, with no clear consensus regarding the temporal nature by which the VML injury immune response is modulated or what potential impact the proposed immune modulation has explicitly on myogenic and fibroadipogenic progenitors within the VML defect.

TA muscle functional improvements were observed with various minced graft iterations, to include the expansion treatment group (50% MG + 50% UBM). Prior studies and the current data have demonstrated that 100% minced graft repair restores approximately half of the torque deficit per given model and these strength improvements are matched by qualitative and quantitative observations of skeletal muscle fiber regeneration [19, 22, 23]. It is therefore noteworthy and was initially promising that the expansion group promoted a similar functional improvement at the 100% MG repair. This level of functional improvement mirrors previous results for a 50% MG repair suspended in collagen hydrogel and a 25% MG repair suspended in a decellularized muscle matrix [23, 31]. However, enthusiasm for these findings is diminished by the concurrent observation that the 50% MG alone also promoted functional recovery to a similar magnitude as the 100% MG repair. A tissue-equivalent control group was not reported in prior expansion studies [31, 50]. Given that acellular biological scaffolds have also been shown to improve functional recovery in the absence of skeletal muscle fiber regeneration [8, 9, 18, 19, 31, 51], functional recovery following 50% MG + 50% UBM, which presented little evidence of muscle fiber regeneration, may similarly result from improvements in efficiency of force transmission. Regardless, these findings indicate that expansion of minced graft-mediated functional recovery is yet to be realized.

## Conclusions

The goal of this study was to evaluate the co-delivery of micronized acellular urinary bladder matrix and minced muscle graft for the treatment of VML with the hope of validating the acellular bioscaffold as an expansion medium for the minced grafts that would allow for their effective implantation at a reduced donor tissue burden. Unfortunately, the micronized UBM impaired skeletal muscle fiber regeneration mediated by minced muscle grafts, likely as a result of exacerbated inflammation. Despite the poor regenerative outcome, the MG expansion product restored approximately half of the functional deficit in this VML model. However, the functional improvements were matched by a 50% MG-only implant, which present superior muscle fiber regeneration and presumably a myogenic-permissive immune response. To that end, the most promising finding herein is that the 50 and 100% MG implants performed similarly in terms of muscle tissue regeneration and functional recovery, signifying a 50% reduction in donor tissue burden in this model without the need of a biomaterial carrier. Significant promise still exists for co-delivery of minced grafts with a myoconductive scaffold, though the current results fall short of supplying an effective expansion product.

## Declarations

The opinions or assertions contained herein are the private views of the authors and are not to be construed as official or as reflecting the views of the Department of the Army or the Department of Defense.
